# First step in developing a 3D biodegradable fibrin scaffold for an artificial ovary

**DOI:** 10.1186/1757-2215-6-83

**Published:** 2013-11-25

**Authors:** Valérie Luyckx, Marie-Madeleine Dolmans, Julie Vanacker, Sarah R Scalercio, Jacques Donnez, Christiani A Amorim

**Affiliations:** 1 Pôle de Recherche en Gynécologie, Institut de Recherche Expérimentale et Clinique, Université Catholique de Louvain, Brussels, Belgium; 2 Gynecology Department, Cliniques Universitaires Saint-Luc, Brussels, Belgium; 3 National Primate Centre, Secretary of Health Policy, Ministry of Health, Belém, Brazil; 4 SRI (Society for Research into Infertility), Brussels, Belgium

**Keywords:** Fibrin, Tissue engineering, Artificial ovary, Ovarian stromal cells, In vitro culture, Scanning electron microscopy

## Abstract

**Background:**

Although transplantation of cryopreserved ovarian tissue is a promising approach to restore fertility in cancer patients, it is not advisable for women at risk of ovarian involvement due to the threat of reintroducing malignant cells. The aim of this study was therefore to find an alternative for these patients by development of an artificial ovary.

**Methods:**

For construction of the artificial ovary matrix, we used a central composite design to investigate nine combinations of fibrinogen (mg/ml) and thrombin (IU/mL) (F/T): F1/T4, F12.5/T1, F12.5/T20, F25/T0.1, F25/T4, F25/T500, F50/T1, F50/T20 and F100/T4. From the first qualitative analyses (handling and matrix size), five combinations (F12.5/T1, F25/T4, F50/T20, F50/T1 and F100/T4) yielded positive results. They were further evaluated in order to assess fibrin matrix degradation and homogeneous cell encapsulation (density), survival and proliferation (Ki67), and atresia (TUNEL) before and after 7 days of in vitro culture. To determine the best compromise between maximizing the dynamic density (Y1) and minimizing the apoptosis rate (Y2), we used the desirability function approach.

**Results:**

Two combinations (F12.5/T1 and F25/T4) showed greater distribution of cells before in vitro culture, reproducible degradation of the fibrin network and adequate support for isolated human ovarian stromal cells, with a high proportion of Ki67-positive cells. SEM analysis revealed a network of fibers with regular pores and healthy stromal cells after in vitro culture with both F/T combinations.

**Conclusion:**

This study reports two optimal F/T combinations that allow survival and proliferation of isolated human ovarian cells. Further studies are required to determine if such a scaffold will also be a suitable environment for isolated ovarian follicles.

## Introduction

While advances in cancer treatment have increased the survival of cancer patients, more long-term side effects can be observed in these patients. Aggressive treatments (particularly alkylating chemotherapeutic agents and total body radiation) are known to cause secondary menopause and infertility. Different options are available to preserve fertility in these young women, but for prepubertal patients or when time does not allow hormonal stimulation, cryopreservation of ovarian tissue is the only feasible option at present. In recent years, ovarian tissue transplantation has proved effective to restore fertility in adults, with 24 live births published to date [1]. However, transplantation of cryopreserved ovarian tissue cannot be safely performed when there is a risk of reimplanting malignant cells. Patients suffering from cancer with a high risk of ovarian involvement, such as leukemia, neuroblastoma or Burkitt lymphoma, are therefore not advised to undergo ovarian tissue transplantation [2]. Indeed, a previous study proved, by PCR analysis and long-term xenografting, malignant cell contamination of cryopreserved ovarian tissue from leukemia patients [3]. This risk may also be present in other types of cancer [2] and for these patients, in whom transplantation of ovarian tissue is not advisable, new options need to be developed.

One alternative that can be proposed is grafting of isolated ovarian follicles [4]. This technique may be considered safe because ovarian follicles are surrounded by a basal membrane, excluding them from the stromal environment, capillaries and nerves [5]. The main challenge is to develop a scaffold which encapsulates these isolated follicles, supporting their survival and growth after transplantation. The structure should maintain the three-dimensional (3D) architecture of ovarian follicles and degrade relatively quickly to allow growth of preantral follicles up to the antral stage. In our research unit, previous studies have demonstrated that isolated human ovarian follicles encapsulated in plasma clots can survive after grafting to immunodeficient mice for one week [4] and develop up to the antral stage after 6 months’ xenografting [6]. Despite these already promising results, we believe we could improve follicle survival and growth and reorganize this artificial ovary into a functioning organ by adding isolated ovarian cells [7]. Indeed, isolated ovarian cells, including stromal and endothelial cells, have demonstrated their primordial role in the formation of a well vascularized and structured ovary-like stroma after one week of grafting [8]. However, plasma clots are difficult to handle, leading to follicle loss.

Fibrin plays an integral part in physiologic blood coagulation. Fibrinogen is a soluble 340 kDa protein that is polymerized into fibrin through the action of thrombin, an active enzyme in the presence of calcium. Factor XIIIa, activated by thrombin, then crosslinks fibrin by the formation of covalent lysyl-glutamine bonds [9-11]. Varying concentrations of fibrinogen and thrombin can influence the morphology and rigidity of the fibrin network, and therefore cell proliferation [12,13]. Numerous research domains have been studying the potential of fibrin gel in tissue engineering. Fibrin can be employed as a vehicle for cell transplantation and delivery of growth factors, hormones or bioactive substances [14]. For instance, transplantation of mesenchymal stem cells in fibrin gel has been studied for cartilage regeneration [15]. Fibrin scaffolds have been developed for future therapeutic strategies in muscular dystrophy, with transplantation of myoblasts, or for bone marrow engineering, with grafting of cord blood-hematopoietic stem cells [16]. Fibrin can be also combined with other substances to promote growth of human amniotic mesenchymal stem cells, in order to improve myocardiac function [17].

Based on these successful studies, we decided to develop a fibrin scaffold that would allow survival and proliferation of isolated human ovarian cells. The aim of the present study was therefore to determine the optimal combination of fibrinogen (F) and thrombin (T) concentrations. Through establishment of an experimental design, we tested different fibrinogen and thrombin combinations (F/T) and analyzed the morphological parameters of fibrin clots. We also used an in vitro culture model with isolated human ovarian stromal cells to evaluate cellular survival and proliferation in the different fibrin clots. This project is the first step in building a 3D biodegradable fibrin scaffold, allowing survival and development of isolated ovarian follicles and ovarian cells.

## Material and methods

### Collection of human ovarian tissue

Use of human ovarian tissue was approved by the Institutional Review Board of the Université Catholique de Louvain (Comité d’Ethique Biomédicale Hospitalo-Facultaire 2012/23MAR/125, N° Enregistrement Belge B403201213872). Ovarian biopsies were obtained from four patients between 26 and 51 years of age (mean ± SD: 44.48 ± 11.80) undergoing laparoscopy for benign gynecological disease. All women agreeing to donate tissue signed the cryopreservation informed consent form. Biopsies were immediately transported on ice to the laboratory in Dulbecco’s modified Eagle’s medium (DMEM) and F12 medium containing L-glutamine and 15 mM HEPES (Gibco, Invitrogen, Merelbeeke, Belgium).

### Ovarian stromal cell isolation procedure

The medullar part of the biopsy was removed with surgical scissors and ovarian cells were isolated using the protocol developed by Vanacker et al. [18]. Briefly, the cortex was cut into 0.5 × 0.5 × 1 mm pieces using a tissue sectioner (McIlwain Tissue Chopper, Mickle Laboratory, Guildford, UK) adjusted to 0.5 mm. The minced human ovarian tissue was then transferred to a conical tube containing 10 ml Dulbecco’s phosphate-buffered saline (PBS) with calcium and magnesium (Lonza, Verviers, Belgium) supplemented with 0.04 mg/ml of Liberase DH (Roche, Vilvoorde, Belgium). Incubation was performed in a water bath at 37°C for 75 min with agitation (120 RPM). The tissue was pipetted every 15 min to mechanically disrupt it. Enzymatic activity was inactivated by adding an equal volume of Dulbecco’s PBS without calcium and magnesium supplemented with 10% fetal bovine serum (FBS, Sigma-Aldrich, Bornem, Belgium). The digested cell solution was successively filtered through 80 μm and 11 μm nylon net filters (Millipore, Brussels, Belgium). After centrifugation, the pellet was re-suspended in 2 mL culture medium (DMEM-F12 containing 10% FBS, 100U/mL penicillin and 100 μg/mL streptomycin), and cells were counted using Trypan Blue (Sigma-Aldrich) and a Bürker chamber (VWR, Leuven, Belgium). The pellet containing ovarian cells was diluted in order to obtain a density of 10,000 cells/cm^2^ in culture flasks.

### In vitro culture of isolated human ovarian stromal cells

Ovarian stromal cells were plated in T-75 plastic flasks (Nunc, Rokskilde, Denmark) and left to adhere for 15 min. Supernatant medium containing cellular debris and non-adherent cells was discarded and replaced with 10 mL fresh culture medium. Culture flasks were incubated in a humidified atmosphere with 5% CO_2_ in air. Culture medium was renewed every 2 days until cellular confluence was achieved. After 22 days of in vitro culture, Accutase® (Sigma-Aldrich) was used for detachment and dissociation of anchorage-dependent cells from the plastic flasks. Cells were counted in a Bürker chamber. After centrifugation, the pellets were resuspended in DMEM-F12 + 10% FBS medium in order to obtain a final concentration of 10,000 ovarian stromal cells/5 μl of medium.

### Formation of fibrin clots

**Figure 1:**
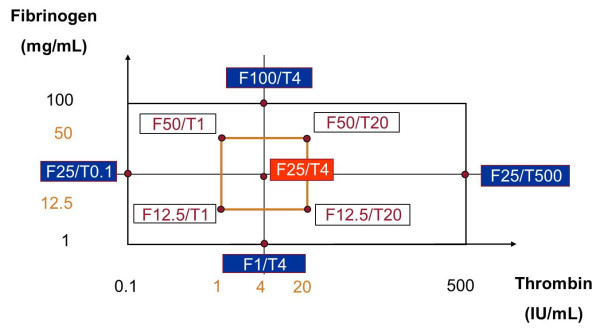
Representation of the different analyzed combinations of fibrinogen and thrombin.

Tissucol is a two-component fibrin sealant provided by Baxter (Braine-l’Alleud, Belgium). When combined, the two components, sealer protein and thrombin, physiologically mimic the final stage of the blood coagulation cascade. The active ingredient in sealer protein (human) is fibrinogen. Fibrinogen (100 mg/mL) was reconstituted in a solution containing 3000KIU/mL of bovine aprotinin, a fibrinolysis inhibitor, at 37°C. Thrombin (500 IU/mL) was reconstituted in 40 mmol/mL of calcium chloride (CaCl_2_) solution. The reconstituted fibrinogen was diluted in saline solution (NaCl 0.9%) (9 g/L of sodium chloride) to various concentrations ranging from 1 to 100 mg/mL, while thrombin was diluted in 40 mmol/L CaCl_2_ solution. The above dilutions yielded five different concentrations of reconstituted fibrinogen, 1 (F1), 12.5 (F12.5), 25 (F25), 50 (F50) and 100 mg/mL (F100), and five concentrations of thrombin, 0.1 (T0.1), 1 (T1), 4 (T4), 20 (T20) and 500 IU/mL (T500). The resulting combinations of the two components are shown in Figure 1.

A droplet of 12.5 μL fibrinogen was placed on a glass petri dish and 50,000 human ovarian cells in 5 μL of medium were mixed with the fibrinogen. This droplet, containing isolated ovarian cells, was mixed with a 12.5 μL volume of thrombin on a plastic petri dish. The resulting fibrin clot was incubated at 37°C for 45 min. After incubation, the clot was gently detached and used for different steps. For each combination, one fibrin clot was embedded in 2% agarose (UltraPure™ Agarose, Invitrogen, Merelbeke, Belgium), fixed in 4% formaldehyde for 24 hours (control) and embedded in paraffin for histological and immunohistochemical studies. Another fibrin clot was fixed in 2.5% glutaraldehyde for future analysis by scanning electron microscopy. Two further fibrin clots were assigned to in vitro culture for one week in the same culture conditions as described above. After one week of in vitro culture, the clots were fixed in 4% formaldehyde and 2.5% glutaraldehyde.

### Histological analysis and cell density

Paraffin-embedded fibrin clots were cut into serial sections of 5 μm. Every fourth section was stained with hematoxylin-eosin (Merck, Darmstadt, Germany) for histological analysis. The other sections were kept for immunohistochemical staining (Superfrost Plus slides, Menzel-Glaser, Braunschweig, Germany).

To evaluate cell density (number of cells per surface unit), 2 or 3 representative sections from fresh controls (day 0) and after in vitro culture (day 7) were examined at 50× magnification, and digitized by means of a Leica DFC295 camera and imaging program (Leica Application Suite, Leica). Using ImageJ, a freely available image-processing and analysis program developed at the National Institutes of Health ( http://rsb.info.nih.gov/ij/), all cells found in sections from fresh controls and after in vitro culture were counted, and analyzed areas of the fibrin clots were measured.

The dynamics of cell density after one week of in vitro culture, expressed in %, was determined by the following equation:

$$\frac{\text{Density}\;\text{day}7‒\text{Density}\;\text{day}0\times 100}{\text{Density}\;\text{day}\;7}$$

### Ovarian stromal cell proliferation

For each F/T combination in different conditions (before and after in vitro culture), one representative slide was immunostained to measure proliferative activity using Ki67 antibody. Sections were deparaffinized with Histosafe (Yvsolab SA, Beerse, Belgium) and rehydrated in 2-propanol (Merck). Endogenous peroxidase activity was blocked by incubating them with 0.3% H_2_0_2_ (Merck) for 30 min at room temperature. After demasking in citrate buffer (pH 6) for 75 min at 98°C, non-specific binding sites were blocked by incubation with normal goat serum for 30 min. The sections were then incubated overnight at 4°C with primary antibody, a mouse monoclonal anti-human Ki67 antibody (M7240, 1:100 dilution, Dako, Glostrup, Denmark). The slides were subsequently incubated for 60 min at room temperature with goat anti-mouse secondary antibody (Dako). Diaminobenzidine (Dako) was used as a chromogen and hematoxylin as a counter-stain. Human proliferative endometrium was used as a positive control for Ki67 labeling. Negative controls consisted of the dilution solution without any anti-human Ki67 antibody. The proliferation index was evaluated as the percentage of Ki67-positive cells for each combination.

### Ovarian stromal cell apoptosis

Apoptosis was analyzed by terminal deoxynucleotidyl transferase-mediated dUTP nick end labeling (TUNEL) to detect DNA fragmentation using the In Situ Cell Death Detection Kit, TMR Red (Roche). The complete protocol was previously described in detail by Martinez-Madrid et al. [19] and Vanacker et al. [20]. The slides were examined under an inverted fluorescence microscope (Leica; Van Hopplynus Instruments, Brussels, Belgium). Red fluorescence was visualized in TUNEL-positive cells by applying an excitation wavelength in the range of 520-560 nm, and observing the emitted light at a wavelength between 570 and 620 nm. Pictures were taken of the sections and ImageJ was used for morphometric analysis of TUNEL-positive surface areas in order to quantify apoptosis. The apoptosis rate was evaluated as the percentage of TUNEL-positive cells for each combination.

### Fibrin degradation

Degradation of the different fibrin formulations was assessed both macroscopically and histologically. After in vitro culture, all clots were measured and observed for the presence of a residual fibrin matrix.

### Scanning electron microscopy (SEM) analysis

After fixation in a 2.5% glutaraldehyde solution, samples were dehydrated successively in baths of different ethanol concentrations (30%, 50%, 70% and 85% ethanol for 15 min each; 100% ethanol for 30 min). The next step involved critical point drying to preserve sample morphology. Using a Balzers CPD030 critical point drier (Bal Tec AG, Fürstentum, Liechtenstein), solvent was progressively replaced with liquid carbon dioxide (CO_2_) thanks to exchange fluids like ethanol. The liquid CO_2_ was then brought to its critical point and converted to the gaseous phase by increasing pressure and temperature. The dried samples were mounted on stubs containing carbon adhesives. Sputter coating with gold (15 nm) was applied to samples with a Balzers Union sputtering device (type 07 120B, Bal Tec AG). The samples were stored at room temperature until analysis with a Jeol 7500-F scanning electron microscope at a resolution of 1.0 nm at 15 kV.

### Experimental design

In order to determine the optimal fibrin formulation for isolated preantral follicles and ovarian cells, namely the optimal combination of thrombin and fibrinogen that maximizes density dynamics (%) and minimizes the apoptosis rate (%), we developed an experimental design. It was based on a central composite design (CCD), which allows a second order (quadratic) model to be constructed for response variable(s). It comprises minimum of nine runs, each run corresponding to a combination of the two factors investigated: four points from the two-level factorial design, four axial points, and at least one run in the center of the design. The center points are usually chosen as the most reliable.

Based on several studies [9,12,13,21], the domain to be covered by the design was set between 1 and 100 mg/ml for fibrinogen and between 0.1 and 500 IU/ml for thrombin. The most promising point was composed of 25 mg/mL fibrinogen and 4 UI/mL thrombin. This combination was chosen because it is very similar to a plasma clot: a low thrombin concentration forming clots with thick fibrin fibers [22] and a higher degradation rate.

Around this central combination, we defined a squared area of interest corresponding to the two-level factorial part of the CCD. The four corresponding runs were named F12.5/T1, F12.5/T20, F50/T20 and F50/T1 (Figure 1).

The four axial points were set as extreme combinations of thrombin and fibrinogen of our domain. Due to asymmetry of the domain on both sides of the theoretical best concentration of thrombin, we had to modify the theoretical axial point of the CCD (Figure 1), so the concentration of thrombin was not symmetrically distributed around the central point.

### Statistical analysis

To determine the best compromise between maximizing density dynamics (Y1) and minimizing the apoptosis rate (Y2), we used the desirability function approach [23]. Each response variable Yi (i = 1, 2) was assigned a desirability function Di that mapped Yi to [0, 1], with Di(Yi) = 0 (response Di(Yi) = 1) when Yi was at a completely undesirable (response completely desirable) value. We chose linear desirability functions. Simultaneous optimization of Y1 and Y2 was then reduced to yield maximization of an overall desirability function D defined using a geometric mean:

$$D=\surd \left[D1\left(Y1\right)+D2\left(Y2\right)\right]$$

where Y1 and Y2 were replaced by their corresponding values Ŷ1 and Ŷ2 obtained from a linear model. The design thus allowed quadratic terms. We further included a random effect to account for correlation between repeated measurements.

## Results

By means of qualitative and quantitative analyses, we aimed to define the best F/T combination that would degrade the fastest and allow proliferation of stromal cells, which in turn would build an adequate environment for ovarian follicles.

### Qualitative analysis of nine fibrinogen/thrombin combinations

In a first step, we analyzed nine F/T combinations established by the experimental design (Figure 1) and selected those that met the following criteria for a reproducible and standard protocol:

• homogeneous fibrin clot formation;

• easy handling of fibrin constructs;

• correct encapsulation of cells.

With F1/T4, fibrin clots were very small in size (less than 1×1 mm) and difficult to manipulate with thin forceps. Fibrin clots from other combinations ranged between 1×5 mm and 6×5 mm in size and were easier to handle. We observed that the volume of fibrin clots increased in correlation with fibrinogen concentrations. With two other combinations (F25/T500 and F12.5/T20), the polymerization of fibrin was too rapid and the clots became stuck inside the micropipette tips during the mixing of the two components. With F25/T0.1, the fibrin clots were fragmented after incubation and detachment from the plastic petri dish. These four F/T combinations did not therefore meet all required criteria. The remaining five combinations (F12.5/T1, F25/T4, F50/T20, F50/T1 and F100/T4) were easily and reproducibly securated and manipulated.

### Quantitative analysis of five fibrinogen/thrombin combinations

#### Cell distribution and fibrin degradation

**Figure 2:**
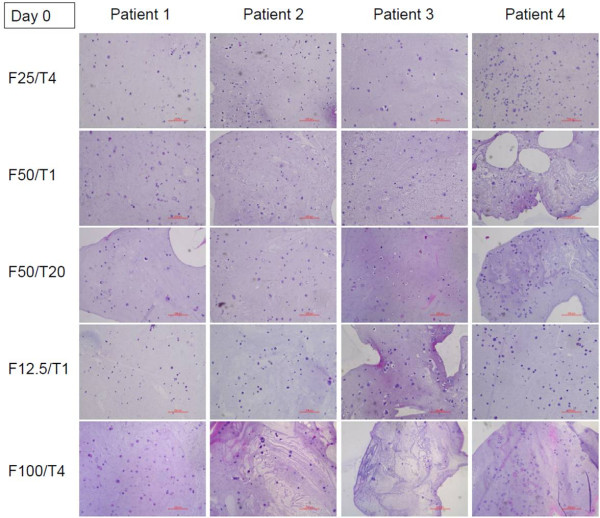
Representative histological section of different fibrin formulations after encapsulation of isolated human ovarian stromal cells from four different patients in a fibrin matrix (day 0).

**Figure 3:**
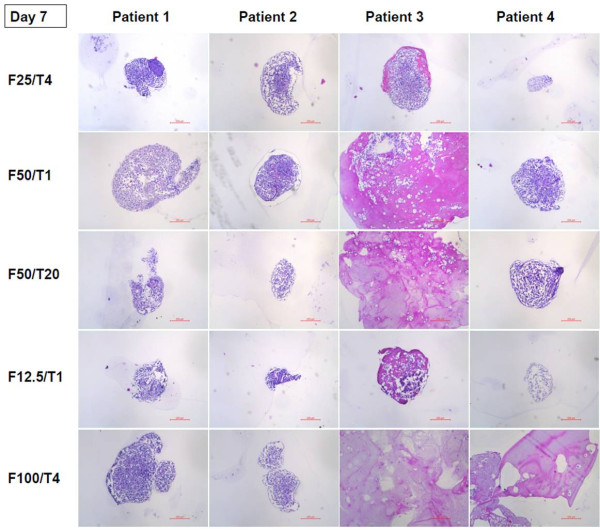
Representative histological section of different fibrin formulations after encapsulation of isolated human ovarian stromal cells from four different patients in a fibrin matrix and after 1 week of in vitro culture (day 7).

Based on the qualitative criteria described above, five combinations were chosen. They were analyzed with quantitative parameters soon after inclusion of isolated human ovarian stromal cells and also after one week of in vitro culture. Histological results on day 0 and day 7 after in vitro culture are shown in Figures 2 and 3, where the distribution of cells inside the fibrin clots can be observed. Macroscopically and histologically, the clots were found to be reduced in volume and size (less than 1×1 mm) after one week of in vitro culture. In some cases, residual fibrin was encountered after culture in histological sections. The most reproducible histological results were obtained with F25/T4 and F12.5/T1 combinations, taking into account the spatial uniformity of cell distribution, homogeneity of the fibrin network and residual fibrin after one week of in vitro culture.

#### Cell density, proliferation and survival after one week of in vitro culture

To quantitatively compare the 5 combinations, cell density was analyzed on day 0 and day 7, and the dynamics of cell density was calculated. These results, together with proliferation and apoptosis indices, are shown in Table 1.

**Table 1:** Results obtained after comparison of 5 combinations of fibrinogen and thrombin with in vitro culture of fibrin clots encapsulating human isolated ovarian stromal cells from four patients (numbered 1 to 4)

**Combination**	**Cell density**		**Density dynamics (%) mean ± SD**	**Proliferation index (%) mean ± SD**	**Apoptotic index (%) mean ± SD**
	**Day 0**	**Day 7**			
**F25/T4**	0.0783 ± 0.0376	2.3038 ± 0.5988	96.46 ± 1.97	4.38 ± 4.81	26.99 ± 23.45
**F50/T1**	0.0524 ± 0.0113	1.8028 ± 1.1627	92.78 ± 9.79	1.35 ± 1.24	18.20 ± 14.94
**F50/T20**	0.0566 ± 0.0248	1.3848 ± 0.8639	86.89 ± 19.60	3.23 ± 2.66	18.50 ± 10.35
**F12.5/T1**	0.0757 ± 0.0148	1.6946 ± 0.2691	95.42 ± 1.28	4.45 ± 2.34	23.19 ± 35.11
**F100/T4**	0.0397 ± 0.0176	0.8243 ± 0.9104	58.29 ± 49.53	5.10 ± 3.40	3.77 ± 1.17

Three different parameters are illustrated: cell density dynamics, proliferation index and apoptosis rate.

For the dynamics of cell density, we rather curiously observed a negative result for one F100/T4 fibrin clot: cell density was better before as opposed to after in vitro culture. For the other combinations, cell density dynamics ranged from 94.2% to 98.9% (F25/T4), 78.1% to 98.0% (F50/T1), 57.5% to 97.8% (F50/T20) and 94.0% to 96.6% (F12.5/T1).

The proliferation index varied between 1.35 ± 1.24% (F50/T1) and 5.10 ± 3.40% (F100/T4). No significant difference was observed between the combinations in terms of proliferation index.

The apoptosis rate also showed great variation for each combination, except for F100/T4. For F12.5/T1, for example, it ranged from 0.9% to 75.6%. The range of the apoptosis index varied between 3.77 ± 1.17% (F100/T4) and 26.99 ± 23.45% (F25/T4).

Statistical analysis, based on the desirability function approach (Harrington, 1965), yielded the following order: (1) F12.5/T1, (2) F25/T4 and F100/T4, (3) F50/T1 and F50/T20.

### Scanning electron microscopy analysis of two fibrinogen/thrombin combinations

**Figure 4:**
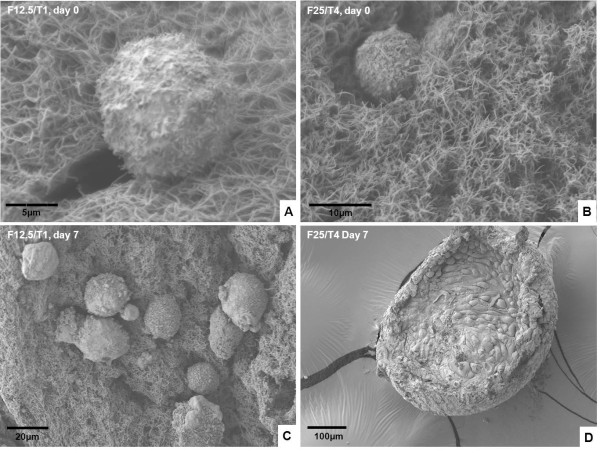
**Illustrations of fibrin clots by scanning electron microscopy (SEM) after encapsulation of isolated human ovarian stromal cells (day 0) (A, B, C) and after 1 week of in vitro culture (day 7) (D).** Two combinations of fibrinogen and thrombin are analyzed: F12.5/T1 **(A, C)** and F25/T4 **(B, D)**.

Based on the results of quantitative analysis of the fibrin clots, we chose two F/T combinations for SEM analysis: F12.5/T1 and F25/T4. SEM was performed to illustrate the surface of the clots, the interaction of isolated cells with fibrin fibers, and the degradation of the clots after in vitro culture. As shown in Figure 4, fibrin clots from both F/T combinations showed a network of thick fibers with regular pores. In both cases, the majority of isolated stromal cells appeared to be healthy in the fibrin network, evidencing some fingering of the membrane. After in vitro culture, fibrin was partially or totally degraded in both F/T combinations, and cells were found to have proliferated up to a point where they were in close contact with each other (Figure 4D).

## Discussion

The aim of this study was to define the optimal F/T combination for the survival and proliferation of isolated human ovarian stromal cells. From the experimental design evaluating nine combinations, five met the criteria for a standardized protocol appropriate for future clinical applications. Of these five combinations, two (F12.5/T1 and F25/T4) showed reproducible degradation of the fibrin network and, based on survival and proliferation analyses, good support for stromal cells, with positive evolution of stromal cell density. Moreover, on SEM, the cells appeared to have successfully colonized the fibrin matrix, which was almost or entirely degraded after seven days of in vitro culture.

### Qualitative analysis of nine fibrinogen/thrombin combinations

The morphology of the fibrin network depends on the conditions of polymerization and concentrations of fibrinogen and thrombin. In our study, we observed that the fibrinogen concentration influenced the size of the fibrin clot. For instance, with the F1/T4 combination, we obtained a tiny fibrin clot, but with F100/T4, a very large matrix. F1/T4 clots were so small that they became extremely difficult to manipulate. Probably due to the small pores produced by large amounts of thrombin [24] and the small final volume size, many cells could not be encapsulated in clots, explaining the high cell population observed in the Petri dish after clot polymerization.

It is also important to mention that the thrombin/fibrinogen ratio is of primary importance because it can influence polymerization. When the ratio is higher than 1, polymerization starts very quickly. For a reproducible protocol in our conditions, the two combinations (F12.5/T20 and F25/T100) with a ratio T/F >1 were too difficult to manipulate. On the other hand, a very low ratio is not desirable as reduced concentrations of thrombin can lower the final level of fibrin [25] and delay fibrin formation [26]. Indeed, we observed that F25/T0.1 fibrin clots were fragmented, possibly because the incubation time was not sufficient for clot formation or due to its flaccidity. Moreover, as a result of this fragmentation, a high number of isolated cells were lost outside the clot. Such loss could also occur with the precious population of isolated preantral follicles.

### Quantitative analysis of five fibrinogen/thrombin combinations

As previously mentioned, quantitative analysis of the remaining five F/T combinations was performed in order to choose the best F/T combination for the artificial ovary by evaluating degradation and the influence of fibrin formulations on cell distribution before in vitro culture, as well as cell behavior after in vitro culture. In all F/T combinations, cells could be found throughout the matrix on day 0. Only with the F100/T4 combination did cell density appear lower than with the other fibrin formulations. This may have been due to clot size, which was increased, with the same number of cells distributed over a larger area.

After seven days of in vitro culture, we observed degradation and shrinkage of all clots. Some of them completely disappeared and only cells could be seen, while in others, a small amount of fibrin was still present around the cells. This was due to fibrinolysis induced by the production of plasminogen activators by stromal cells [27,28] and the absence of aprotinin in the culture medium that could have inhibited this process [9].

Cell density dynamics showed positive evolution of the cell population in our fibrin matrices. Although no statistical difference was found between fibrin formulations, there was a striking numerical difference in the F100/T4 combination, where the density dynamics was lower than in other combinations. This could have been caused by the higher fibrinogen concentration and dispersion of the cells on day 0.

The proliferation index was also similar between the various fibrin formulations. Several studies report that the combination of different fibrinogen and thrombin concentrations appears to have an impact on cell proliferation [12,29-31]. For human mesenchymal stem cells studied for bone tissue regeneration, Bensaid et al. [29] observed adhesion, spread and proliferation of cells for fibrin scaffolds with a fibrinogen concentration not higher than 18 mg/ml. Cox et al. [30] showed that fibroblasts proliferated well within 3-D fibrin clots containing low fibrinogen (5-17 mg/mL) and thrombin (1-167 U/mL) concentrations. According to Duong et al. [9], fibroblasts prefer to grow and typically exhibit a spread morphology in lower formulations of fibrinogen and thrombin that yield lower kPa values for stiffness modulus. All these observations appear to validate our choice of fibrin formulations, with lower fibrinogen concentrations (F12.5/T1 and F25/T4).

Interestingly, in the F12.5/T1 and F25/T4 combinations, a high apoptosis rate was also observed. We speculate that when a critical point is attained, cell death increases because of the confined space of the fibrin matrix. At this moment, cells could continue multiplying, but to the detriment of other cells.

### Scanning electron microscopy analysis of two fibrinogen/thrombin combinations

Since F12.5/T1 and F25/T4 combinations offered the most reproducible results, they were analyzed by SEM. On day 0, both fibrin formulations showed thick fibers and regular pores, which can be explained by the mechanism involved in fibrin clot formation. Fiber diameter is determined by a balance between the rate of lateral aggregation of monomers and the rate of fibrinopeptide cleavage [32]. The thrombin concentration present during clot formation is directly related to the rate of fibrinopeptide cleavage [32]. Therefore, at low thrombin concentrations, the rate of fibrinopeptide cleavage is reduced, allowing larger lateral aggregation of protofibrils and production of thicker fibers.

After in vitro culture, SEM images showed healthy cells in close contact with each other due to their proliferation and degradation of the fibrin matrix. Chiu et al. [11] demonstrated that fibrinogen and thrombin concentrations have an inverse relationship with the overall porosity, pore size and, hence, fluid permeability of fibrin matrices. Lower concentrations of fibrinogen found in both these F/T combinations possibly allowed greater diffusion of nutrients, growth factors and waste removal, which positively affected cell proliferation.

## Conclusions

In conclusion, this study indicates that fibrin formulations with low fibrinogen and thrombin concentrations are a promising option for the construction of a biodegradable and biocompatible 3D matrix for isolated ovarian stromal cells, allowing their survival and proliferation. Our experimental design yielded two combinations that showed encouraging results. The next step will involve evaluation of the survival and development of isolated preantral follicles encapsulated in these fibrin formulations in order to assess if they can be used as a matrix to construct an artificial ovary.

## Competing interests

The authors declare that they have no competing interests.

## Authors’ contributions

VL and CAA designed study, performed experiments, wrote the manuscript and interpreted data. JV and SRS performed experiments. M-MD and JD collected biopsies and corrected the manuscript. All authors read and approved the final manuscript.

## Authors’ information

Where the work was performed: Université Catholique de Louvain, Pôle de Recherche en Gynécologie, Institut de Recherche Expérimentale et Clinique, Brussels, Belgium.
